# NSOM/QD-Based Visualization of GM1 Serving as Platforms for TCR/CD3 Mediated T-Cell Activation

**DOI:** 10.1155/2013/276498

**Published:** 2013-10-30

**Authors:** Liyun Zhong, Zhun Zhang, Xiaoxu Lu, Shengde Liu, Crystal Y. Chen, Zheng W. Chen

**Affiliations:** ^1^Laboratory of Nanophotonic Functional Materials and Devices, South China Normal University, Guangzhou, Guangdong 510006, China; ^2^Department of Microbiology & Immunology, Center for Primate Biomedical Research, University of Illinois at Chicago, Chicago, IL 60612, USA

## Abstract

Direct molecular imaging of nanoscale relationship between T-cell receptor complexes (TCR/CD3) and gangliosidosis GM1 before and after T-cell activation has not been reported. In this study, we made use of our expertise of near-field scanning optical microscopy(NSOM)/immune-labeling quantum dots- (QD-)based dual-color imaging system to visualize nanoscale profiles for distribution and organization of TCR/CD3, GM1, as well as their nanospatial relationship and their correlation with PKC**θ** signaling cascade during T-cell activation. Interestingly, after anti-CD3/anti-CD28 Ab co-stimulation, both TCR/CD3 and GM1 were clustered to form nanodomains; moreover, all of TCR/CD3 nanodomains were colocalized with GM1 nanodomains, indicating that the formation of GM1 nanodomains was greatly correlated with TCR/CD3 mediated signaling. Specially, while T-cells were pretreated with PKC**θ** signaling inhibitor rottlerin to suppress IL-2 cytokine production, no visible TCR/CD3 nanodomains appeared while a lot of GM1 nanodomains were still observed. However, while T-cells are pretreated with PKC**α** **β** signaling inhibitor GÖ6976 to suppress calcium-dependent manner, all of TCR/CD3 nanodomains were still colocalized with GM1 nanodomains. These findings possibly support the notion that the formation of GM1 nanodomains indeed serves as platforms for the recruitment of TCR/CD3 nanodomains, and TCR/CD3 nanodomains are required for PKC**θ** signaling cascades and T-cell activation

## 1. Introduction

TCR/CD3 nanodomains play a crucial important role in T-cell activation, including signal transduction, membrane trafficking, cytoskeletal organization, adhesion, migration and pathogen entry [1–8]. To date, it has been demonstrated that the formation of TCR/CD3 nanodomain is a precondition to induce the immunologic synapse, in which a variety of signaling molecules are recruited to TCR/CD3 nanodomains leading to its signaling amplifies and facilitating T-cell activation [6, 9]. Though it was reported that lipid rafts play a integral role in the synapse formation between antigen presenting cells and T-cells [10], and GM1, a mono-sialoganglioside and glycosphingolipid, which is the main component of lipid rafts and can be recognized by cholera toxin, has been employed as the lipid raft marker [11, 12], but whether GM1 clustering is required for the recruitment of TCR/CD3 still remains controversial [13, 14]. Furthermore, although protein kinase C theta (PKC*θ*) isoform has been identified as a specific constituent of signaling cascades in TCR/CD3 mediated activation events [15–17], whether TCR/CD3 nanodomains are correlated with the functional ability of TCR/CD3 complex to initiate PKC*θ* signaling cascades is kept unknown. Now, direct molecular imaging of nanospatial relationship between TCR/CD3 and GM1 before and after T-cells activation has not been reported. Scanning fluorescence nanoscale visualization of each of these molecules on cell surface is indeed lacking. Some studies of TCR/CD3 nanodomains interaction with GM1 using conventional techniques such as fluorescence/confocal microscopy or indirect biochemistry analyses of membrane rafts appear to be inconclusive with conflicting results [10, 11, 18]. However, the interaction between TCR/CD3 and GM1 for achieving T-cell activation has not been directly imaged at nanoscale.

We have recently innovated the use of near-field scanning optical microscopy (NSOM) [19, 20] and quantum dots (QD) based nanotechnology through dipole-polarization and dual-color to visualize nanoscale distribution and organization of antigen-specific TCR/CD3, coreceptor CD4, CD8, and nanospatial relationship between TCR/CD3 and CD4 or CD8 in sustained activation of primary T-cells [21, 22]. In the current study, we intend to directly visualize nanoscale nanospatial relationship between TCR/CD3 and GM1 before and after T-cells activation, moreover to detect whether GM1 clustering or TCR/CD3 nanodomains are required for PKC*θ* signaling cascades and T-cell activation.

## 2. Results 

### 2.1. NSOM/QD-Based Imaging Revealed That GM1 Clustering Serves as Platforms for the Recruitment of TCR/CD3 Nanodomains

Although it has been well demonstrated that one of imaging features for TCR/CD3 mediated T-cell activation is TCR/CD3 clustering at the center of the T-cell/antigen presentation cell (APC) interface [23], our recent work also showed that Ag-specific TCR/CD3 was clustered and colocalized with co-receptor CD4 or CD8 to form nanodomains or micro-domains during Ag-induced activation of T-cells [9, 21]. Moreover, it was reported that GM1 rich domains participate in the signaling cascades for TCR/CD3 mediated activation [24]. We therefore ask whether GM1 was clustered and colocalized or interacted with TCR/CD3 after anti-CD3/anti-CD28 Ab costimulation. 

**Figure 1:**
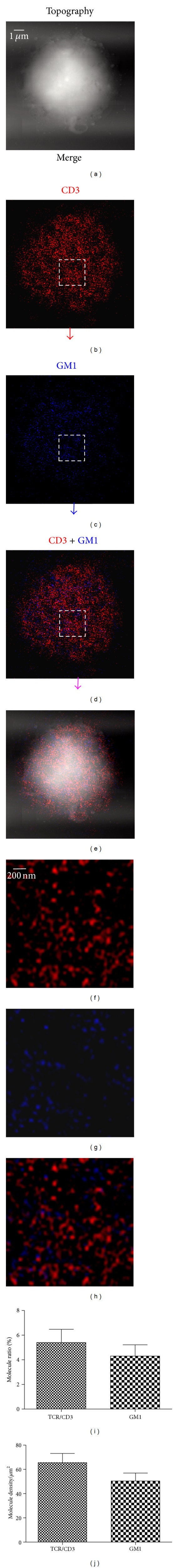
NSOM/QD-based dual-color imaging of CD3 and GM1 for unstimulated T-cells. (a) T-cell topography (b) Fluorescence image of QD-bound CD3 (red). (c) Fluorescence image of QD-bound GM1 (blue). (d) Two color fluorescence merge image. (e) Topography-fluorescence merge image. ((f), (g) and (h)) Zoom images of the areas as indicated by the squares on (b), (c) and (d), respectively. (i) The percentage numbers of TCR/CD3 or GM1 molecules that are arrayed to form nanodomains. (j) Molecule density of TCR/CD3 nanodomains or GM1 nanodomains. Scale bars are 1 *μ*m for (a), (b), (c), (d) and (e) and 200 nm for (f), (g) and (h).

**Figure 2:**
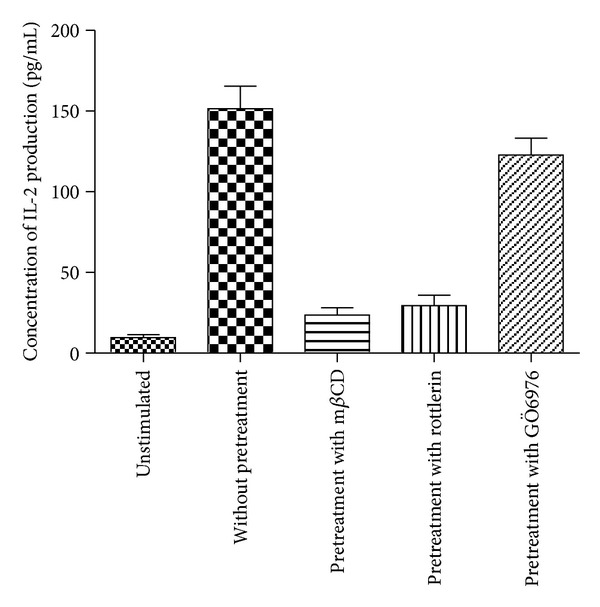
IL-2 concentration of T-cells costimulated with anti-CD3/anti-CD28 Ab for unstimulated, without pretreatment and pretreatment by mCD or Rottlerin or GÖ6976, respectively.

**Figure 3:**
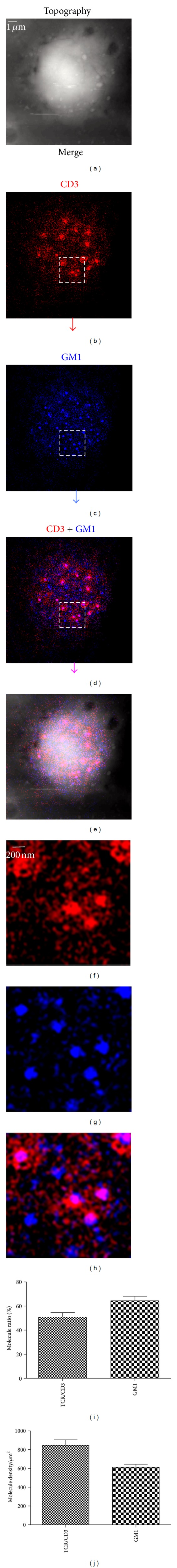
NSOM/QD-based dual-color imaging of CD3 and GM1 for T-cells costimulated with anti-CD3/anti-CD28 Ab. (a) T-cell topography, (b) fluorescence image of QD-bound CD3 (red). (c) Fluorescence image of QD-bound GM1 (blue). (d) Two color fluorescence merge image. (e) Topography-fluorescence merge image. ((f), (g), and (h)) Zoom images of the areas as indicated by the squares on (b), (c) and (d), respectively. (i) The percentage numbers of TCR/CD3 or GM1 molecules that are arrayed to form nanodomains. (j) Molecule density of TCR/CD3 nanodomains or GM1 nanodomains. Scale bars are 1 *μ*m for (a), (b), (c), (d) and (e) and 200 nm for (f), (g) and (h).

As an initial effort to directly visualize nanoscale distribution of TCR/CD3 and GM1 before T-cells activation, we first simultaneously imaged CD3 and GM1 on cell surface of unstimulated T-cells using NSOM/QD based dual-color imaging system. As we recently described [9], most of CD3 and visible GM1 were detected as 70–110 nm nanoclusters (equivalent to 2–4 QD fluorescence intensity and size) and distributed randomly on the surface of unstimulated T-cells (Figure 1), accordingly, the concentration of IL-2 cytokine production (9.6 ± 1.3 pg/mL) is very low (Figure 2). To facilitate evaluation of nanoscale distribution and organization of TCR/CD3 and GM1, nanostructures of these molecules were defined based on the nano-concept and immunofluorescence sizes of them: (i) ≤200 nm nanoclusters, (ii) >200 but <500 nm nanodomains, and (iii) ≥500 nm microdomains. Interestingly, after the anti-CD3/anti-CD28 Ab costimulation, both TCR/CD3 and GM1 underwent changes in nanospatial distribution and formed well-organized nanodomains on the cell surface, and the corresponding concentration of IL-2 cytokine production was increased significantly (151.4 ± 14.6 pg/mL) (Figure 2), Moreover, though the size of GM1 nanodomains (200–300 nm) (Figures 3(c) and 3(g)) was significantly smaller than that of TCR/CD3 nanodomains (200–500 nm) (Figures 3(b) and 3(f)) and the average molecule density of GM1 in nanodomains (612 ± 27/*μ*m^2^) was less than that of TCR/CD3 (847 ± 59/*μ*m^2^) (Figure 3(i)), but the percentages of GM1 molecules arrayed to form nanodomains (64.4 ± 4.6%) are higher than of TCR/CD3 nanodomains (50.9  ±  3.8%) (Figure 3(j)). Importantly, merge NSOM showed that all of TCR/CD3 nanodomains were colocalized with GM1 nanodomains (Figures 3(d) and 3(h)), suggesting that so many GM1 molecules clustering and colocalization with TCR/CD3 clustering should play a important role in the recruitment of TCR/CD3 nanodomains.

**Figure 4:**
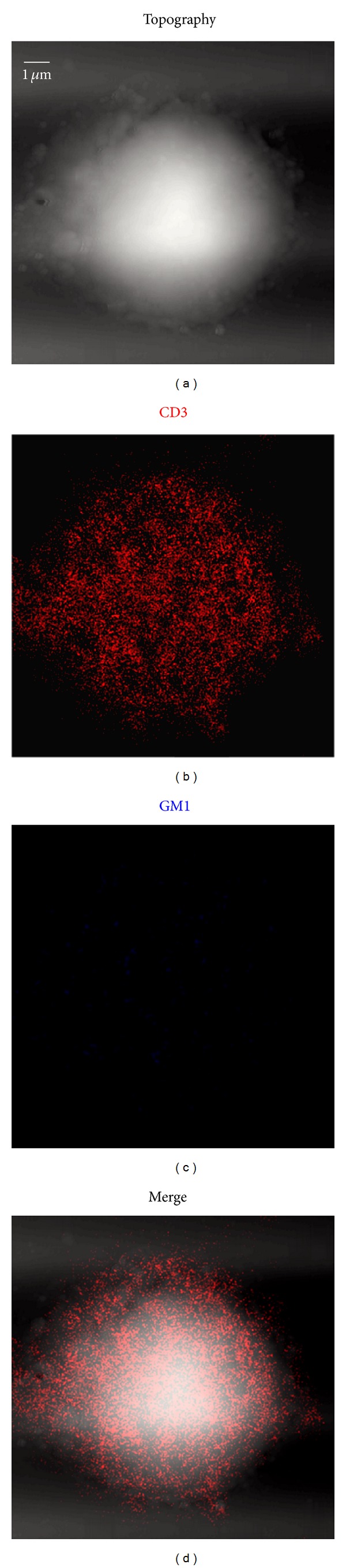
NSOM/QD-based dual-color imaging of CD3 and GM1 for T-cells costimulated with anti-CD3/anti-CD28 Ab after methyl-*β*-cyclodextrin pretreatment.

To determine whether GM1 clustering is required for the recruitment of TCR/CD3 nanodomains, T-cells were pretreated with methyl-beta-cyclodextrin (m-*β*-CD) to destroy the lipid raft integrity. Interestingly, after anti-CD3/anti-CD28 Ab costimulation, no visible TCR/CD3 nanodomains appeared and most of TCR/CD3 were distributed randomly on the cell surface (Figure 4(b)), and moreover, the corresponding concentration of IL-2 cytokine production was greatly decreased (23.6 ± 3.2 pg/mL) (Figure 2). Clearly, it should be reasonable to think that GM1 clustering as nanodomains serves as platforms for the recruitment of TCR/CD3 nanodomains.

### 2.2. NSOM/QD-Based Imaging Reevealed That TCR/CD3 Nanodomains Were Required for PKC*θ* Signaling Cascades

**Figure 5:**
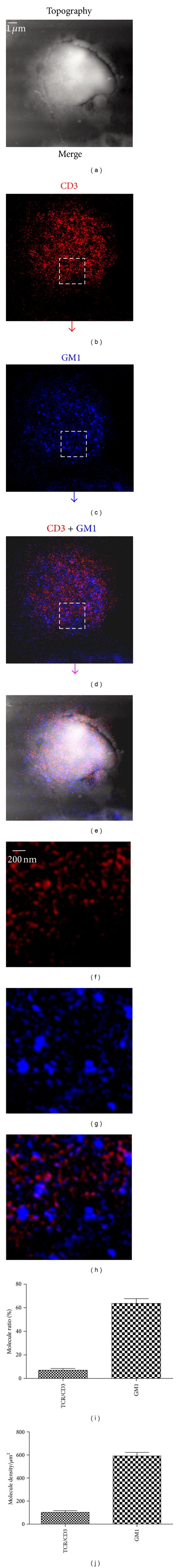
NSOM/QD-based dual-color imaging of CD3 and GM1 for T-cells costimulated with anti-CD3/anti-CD28 Ab after PKC*θ* inhibitor Rottlerin pretreatment. (a) T-cell topography, (b) fluorescence image of QD-bound CD3 (red). (c) Fluorescence image of QD-bound GM1 (blue). (d) Two color fluorescence merge image. (e) Topography-fluorescence merge image. ((f), (g), and (h)) Zoom images of the areas as indicated by the squares on (b), (c) and (d), respectively. (i) The percentage numbers of TCR/CD3 or GM1 molecules that arrayed to form nanodomains. (j) Molecule density of TCR/CD3 nanodomains or GM1 nanodomains. Scale bars are 1 *μ*m for (a), (b), (c), (d) and (e) and 200 nm for (f), (g) and (h).

Although it has demonstrated that protein kinase C*θ*(PKC *θ*), which is a novel PKC isoform and appears to be a specific constituent of TCR/CD3 mediated signaling cascades, was associated with the formation of immunological synapse and IL-2 cytokine production during T-cells activation [16]. Therefore, we ask whether the formation of TCR/CD3 nanodomains was associated with PKC*θ* signaling cascade. To address this, in this study, T-cells were pretreated with PKC*θ* inhibitor rottlerin to suppress the concentration of IL-2 cytokine production. Interestingly, after anti-CD3/anti-CD28 Ab costimulation, no visible TCR/CD3 nanodomains can be observed and TCR/CD3 are distributed randomly (Figures 5(b) and 5(f)) while some of GM1 nanodomains were still observed on cell surface (Figures 5(c) and 5(g)), accordingly, the concentration of IL-2 cytokine production of T-cells pretreated with Rottlerin was decreased to 29.4 ± 6.2 pg/mL, which was significantly lower than that without pretreatment T-cells (Figure 2), and moreover, the percentages of TCR/CD3 that are arrayed to form nanodomains (7.0 ± 0.8%, Figure 5(i)) and the average molecule density of TCR/CD3 in nanodomains (103 ± 14/*μ*m^2^, Figure 5(j)) were greatly less than those without pretreatment T-cells (Figures 3(i) and 3(j)). However, the percentages of GM1 that arrayed to form nanodomains (63.7 ± 5.6%, Figure 5(i)) and the average molecule density of GM1 in nanodomains (592 ± 31/*μ*m^2^, Figure 5(j)) were nearly the same with those without pretreatment T-cells (Figures 3(i) and 3(j)), respectively, suggesting that intrinsic capability of formation of TCR/CD3 nanodomains would implicate a positivefeedback or self-enhancing mechanism since strengthening TCR/CD3 interaction with other signaling molecules would promote downstream PKC*θ* signaling cascades. 

**Figure 6:**
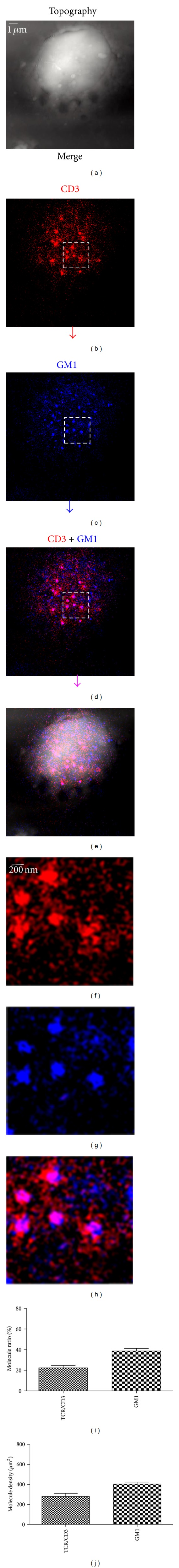
NSOM/QD-based dual-color imaging of CD3 and GM1 for T-cells costimulated with anti-CD3/anti-CD28 Ab after PKC*αβ* inhibitor GÖ6976 pretreatment. (a) T-cell topography. (b) Fluorescence image of QD-bound CD3 (red). (c) Fluorescence image of QD-bound GM1 (blue). (d) Two color fluorescence merge image. (e) Topography-fluorescence merge image. ((f), (g) and (h)) Zoom images of the areas as indicated by the squares on (b), (c) and (d), respectively. (i) The percentage numbers of TCR/CD3 or GM1 molecules that arrayed to form nanodomains. (j) Molecule density of TCR/CD3 nanodomains or GM1 nanodomains. Scale bars are 1 *μ*m for (a), (b), (c), (d) and (e) and 200 nm for (f), (g) and (h).

In contrast, T-cells were also pretreated with protein kinase C alpha and beta (PKC*αβ*) inhibitor GÖ6976 to suppress calcium-dependent manner. Interestingly, after anti-CD3/anti-CD28 Ab co-stimulation, though both GM1 nano-domains and TCR/CD3 nano-domains still were observed (Figures 6(b), 6(c)), but their amount were greatly less than those without pretreatment T cells (Figures 3(b), 3(c)) The percentages of TCR/CD3 and GM1 that arrayed to form nanodomains were, respectively, 22.4  ±  2.1% and 38.7  ±  2.6% (Figure 6(i)) and the corresponding molecule density in nanodomains was, respectively, 280 ± 41/*μ*m^2^ and 405 ± 22/*μ*m^2^ (Figure 6(j)); accordingly, the concentration of IL-2 cytokine production of T-cells pretreated with GÖ6976 was decreased to 122.8 ± 12.6 pg/mL which were significantly lower than those without pretreatment T-cells (Figures 3(i), 3(j), and 2). But importantly, NSOM merge image showed that all of TCR/CD3 nanodomains were still colocalized with GM1 nanodomains (Figures 6(d) and 6(h)), indicating that GM1 was associated with the classic PKC*αβ* signaling cascades. 

Collectively, the above results provided evidence demonstrating that GM1 clustering indeed serves as platforms for the recruitment of TCR/CD3 nanodomains, the formation of TCR/CD3 nanodomains indeed is required for facilitating interaction between TCR/CD3 and other signaling molecules, and thus enhances TCR/CD3 mediated PKC*θ* signaling cascades. 

## 3. Conclusion 

NSOM/QD based dual-color image directly revealed the nanospatial relationship between TCR/CD3 and GM1 during T-cell activation. While confocal fluorescence microscopy and FRET show TCR/CD3 colocalized with GM1 during T-cell activation, there is actually no nanoscale information conceiving whether and how each of these molecules is distributed and shared nanospace on membrane. Now, the NSOM/QD-based nanoscale imaging system allowed us to directly visualize distribution and organization of these molecules on cell-membrane surface.

A new and interesting observation from our novel NSOM/QD is based dual-color imaging studies is that the formation of TCR/CD3 nanodomains are greatly associated with PKC*θ* signaling cascades. Since PKC*θ* is an essential component for inducing TCR/CD3 mediated signaling and IL-2 cytokine production, it should be reasonable to think that formation of TCR/CD3 nano-domains may involve the cytoskeletal rearrangement and the recruitment of various signaling molecules into the immunological synapse, and thus would enhance ZAP70 phosphorylation and T-cell activation. In contrast, the formation of GM1 nanodomains is associated with PKC*αβ* signaling cascades, and while the amount of GM1 nanodomains is decreased, the amount and size of TCR/CD3 nanodomains are remarkably decreased, suggesting that GM1 clustering serve as platforms for the recruitment of TCR/CD3 nanodomains, and enhance TCR/CD3 mediated PKC*θ* signaling cascades and T-cell activation. Importantly, this NSOM/QD-based dual color imaging system provides a powerful tool to directly visualize nanoscale profiles for distribution and organization of cell-surface molecules on cell membrane and enable better understanding of distribution-function relationship.

## 4. Materials and Methods

### 4.1. Samples and Reagents

Human peripheral blood mononuclear cells (PBMCs) were obtained from three healthy volunteers. Anti-CD3-coated 96-well plates were obtained from BD Biosciences. RPMI-1640 culture medium was obtained from GibcoBRL Corp. Rabbit anti-human CD3 was from Dako, Methyl-*β*-cyclodextrin (m-*β*-CD) and Biotinylated anti-human cholera toxin B was obtained from Sigma, Biotinylated anti-mouse IgG was from Invitrogen, Anti-rabbit IgG (H+L)-conjugated quantum dot (QD) 655 and streptavidin-conjugated QD 605 were from Invitrogen. QDs were centrifuged and filtered as previously described to remove aggregates of QDs [21, 22]. PKC*θ* inhibitor Rottlerin and PKC*αβ* inhibitor GÖ6976 were obtained from Calbiochem (CA, USA).

### 4.2. Treatment and Activation of Cells

Peripheral blood was collected from health volunteers. PBMCs were separated by Ficoll-Hypaque gradient centrifugation and washed with phosphate-buffered saline (PBS) as described by our previous reports [7]. For the anti-CD3 Ab stimulation, PBMCs at a cell density of 2 × 10^5^ cells/mL were seeded into anti-CD3 Ab-coated 96-well plates for 48-hour culture in RPMI 1640 containing 10% FBS at 37°C in a 5% CO_2_ atmosphere. For the anti-CD3/anti-CD28 Ab costimulation, PBMC at a cell density of 2 × 10^5^ cells/mL were seeded onto anti-CD3 Ab-coated 96-well plates and cocultured with 5 ng/mL anti-CD28 Ab for 48 hours in RPMI 1640 containing 10% FBS at 37°C in a 5% CO_2_ atmosphere. 

To investigate the role of lipid raft in T-cells activation, PBMCs at a cell density of 1 × 10^6^ cells/mL were pretreated by methyl-*β*-cyclodextrin (m-*β*-CD) to destroy lipid raft integrity [14]. Moreover, to study the role of PKC*θ* for TCR/CD3 mediated signaling, PBMCs at a cell density of 1 × 10^6^ cells/mL were pretreated with or without the PKC*θ* inhibitor Rottlerin (3 *μ*M) or PKC*αβ* inhibitor G GÖ6976 (2 *μ*M) at 37°C for 30 min and centrifuged. In addition, to compare the activity of different treatment T-cells, IL-2 cytokine production was detected in supernatant by ELISA as the manufacturer's instruction (Bender Medsystems, Austria). 

### 4.3. Immune Staining

For T-cells immunolabeling, 2% formalin/PBS solution was first used to fix T-cells to rule out the possibility of non-specific activation of T-cell which is induced by antibody labeling. In the first color labeling, biotinylated anti-human Cholera Toxin B antibody was used to label GM1 molecules, followed by QD streptavidin conjugated 605. In the second color labeling, rabbit anti-human CD3 was used to label CD3, followed by anti-rabbit QD IgG (H+L) conjugated 655. Finally, 2% formalin/PBS solution was used again to further fix the cells. For the above each labeling step, FBS/PBS was applied to wash twice to remove any unbound antibody or QDs. For NSOM imaging study, dd water suspensions of cells were spread onto glass cover slides that were pretreated with poly-L-lysine (Sigma) and air dried at room temperature for NSOM imaging. Control labeling with isotype control antibody was performed simultaneously to rule out the possibility of non-specific labeling, as described previously [21]. Ab- or streptavidin-conjugated QD appear to have sizes of *≈*25 nm [21]. (CD3 complex and GM1 may have nanospatial sizes of ~10 nm and ~1–5 nm, resp.).

### 4.4. NSOM Imaging

An Aurora-3 NSOM system (Veeco) was used in this study. The system was shown schematically in our pervious study [22]. The continuous wave semiconductor laser (Coherent, USA; cube, 404 nm) was launched into a single mode optical fiber (Thorlabs Inc., USA) and used as excitation source. Straight, aluminum-coated probe (Veeco) with an aperture diameter of 50–80 nm was used for imaging. It should be noted that no significant difference in full width at the half maximum (FWHM) of fluorescent spots when we used different probes [21, 22]. The probe tip was attached to piezoelectric quartz tuning fork (resonance frequency ~93 KHz), and probe-sample distance was maintained constant of 10 nanometers by tuning-fork-based shear-force feedback. This mode of operation provided simultaneous topographic and optical data, which was collected with a 40x, NA 0.65 objective (Olympus, Japan), split into two beams by a cube beamsplitter (Newports Inc., USA), and then detected by two APDs (PerkinElmer, Canada) in 0° and 90°, respectively. Optical filters 655 ± 10 nm and 605 ± 10 nm (Newports Inc., USA) were used to separate the fluorescence from the excitation light and the background. The samples were mounted onto the XY stage with full scanning range of 30 *μ*m × 30 *μ*m, and a video camera was used to locate the regions of interest. The images were stable and reproducible during repeated scanning. In this study, the laser excitation intensity was 120 W/cm^2^, the images consisted of 400 × 400 measured points, the integration time for all the images was 30 ms, and most images have been slightly low-pass filtered.

### 4.5. Image Processing, Data Analyses and Statistics

SPMLAB 6.02 software (Veeco) was used to obtain high-quality NSOM fluorescence image and topographic image by leveling and convolution. MATLAB 7.0 was used for the following image processing and analyses. Firstly, two fluorescence images denoted two labeled molecules (CD3 and GM1) and acquired simultaneously from NSOM were color-coded in red and blue, respectively, and then the 2-dimensional merged image of two color fluorescence images was obtained by the intensity superposition algorithm of point to point. MATLAB 7.0 was also used to calculate the fluorescence intensity and measure FWHM distribution of fluorescent spots. The number of QD molecules in each fluorescence spot was estimated based on the fluorescence intensity of single QD (see below), whereas the intensity of each spot was determined by adding all photon counts with a contour of 15% of the peak intensity. For the molecular density determination, the fluorescence intensity of fluorescent spots was analyzed to determine the average fluorescence signal representing the average QD numbers. At the excitation laser intensity of 120 W/*μ*m^2^, a typical count rate for individual QD655 and QD605 was *≈* 7,000 counts/second and *≈*4,500 counts/second, respectively (these values were reproducible in repeat experiments). And then the QD numbers were used to correlate the molecule numbers based on the conservative assumption that the QD : secondary Ab : primary Ab : target molecule = 1 : 1 : 1 : 1 [21, 22]. And then the molecular density was determined by dividing the molecule numbers over the nanodomains areas. Student *t*-test was used to calculate the *P* value, as described previously [22], to determine the statistical difference of molecular density or the percentages of molecules localized into nanodomains after different stimulations.

## Abbreviations

NSOM: Near-field scanning microscopy
QD: Quantum dot
TCR: T-cell Receptor
PKC*θ*: Protein kinase C theta
PKC*αβ*: Protein kinase C alpha and beta
m-*β*-CD: Methyl-beta-cyclodextrin.
